# Molecular studies of rust on European aspen suggest an autochthonous relationship shaped by genotype

**DOI:** 10.3389/fpls.2023.1111001

**Published:** 2023-02-20

**Authors:** Abu Bakar Siddique, Laura Menke, Melis Dinedurga, Benedicte Riber Albrectsen

**Affiliations:** Department of Plant Physiology, Umeå Plant Science Centre (UPSC), Umeå University, Umeå, Sweden

**Keywords:** *Melampsora*, pathogen, *Populus tremula*, genotype effect, condensed tannins, surveillance, biomonitoring, PCR

## Abstract

Forests are at increasing risk from pathogen outbreak. Climate change for example enhance the risk of local disease outbreaks, and naturalization of exotic pathogens may follow human activities, warranting robust pest surveillance routines to support forest management. Melampsora pinitorqua (pine twisting rust) is of concern in Swedish forestry, and here we evaluate the use of visible rust scores (VRS) on its obligate summer host, European aspen (Populus tremula) as a tool for quantification of the pathogen. With use of species-specific primers, we could detect the native rust, but we failed to detect two exotic rusts (M. medusae and M. larici-populina). We found that aspen genotype determined the presence of fungal genetic markers (amplifying the ITS2 region of the fungal rDNA sequence) as well as DNA sequences specific to M. pinitorqua. We correlated VRS with the amount of fungal DNA in the same leaf, and we related the findings to aspen genotype-specific parameters such as the ability to synthesize and store leaf condensed tannins (CT). At the genotype level both positive and negative relationships were observed between CTs, fungal markers, and rust infestations. However, at the population level, foliar CT concentrations correlated negatively with general fungal- and rust-specific marker abundances. Our results, therefore, do not support the use of VRS to assess Melampsora infestation in Aspen. They do, however, suggest that the relationship between European aspen and rust infestation may be characterized as autochthonous in northern Sweden.

## 1 Introduction

Forests increasingly suffer from pests and pathogens that negatively affect tree growth and productivity (Linnakoski et al., 2019). In Sweden, forests may be owned by private households, companies, or the state, but a degree of homogeneity in forest management is maintained through strict supervision by the national Swedish Forest Agency. Coniferous trees are prioritized for production in northern Sweden. However, the native Scots pine (*Pinus silvestris*) is damaged by twisting rust (*Melampsora pinitorqua*), which uses the European aspen (*Populus tremula*) as an obligate alternate host. Aspen is a highly regenerative and fast-growing early succession deciduous tree (Kassfeldt, 2009) that is rich in phenolic defense compounds including condensed tannins (CTs), which are considered mediators of stress tolerance (Ullah et al., 2017; Harding, 2019). Condensed tannins are bioactive phenolic polymers that vary in concentration with both biotic and abiotic factors including season, exposure, and host age (Rubert-Nason and Lindroth, 2021). Moreover, recent studies point at fast responses in tannin concentrations in response to fungal infestation (Ullah et al., 2019; Chowdhury et al., 2023). Leaf CTs thus vary considerably within a genotype, but within populations, high and low CT-producers may be distinguished (e.g. Decker et al., 2017; Bandau et al., 2021).

*Melampsora* rusts are biotrophic pathogens that alter physiological and metabolic processes in the hosts. Consequently, rust infection stunts aspen growth and reduces the value of pine timber (Tabor et al., 2000; May-De Mio and Ruaro, 2008; Toome et al., 2009; Cortizo, 2014; Gortari et al., 2018a; Gortari et al., 2018b). Rust fungi produce bright orange urediniospores that cause seasonal rust outbreaks in natural aspen stands and poplar plantations throughout Europe (Barrès et al., 2012). The risk of rust infection in pines has been linked to proximity to aspen stands, genotype, and soil fertility (Mattila, 2005). Therefore, during the 1960s and 1970s, Swedish forests were subjected to aerial treatments with herbicides to eradicate the less valuable aspen (Eidmann and Klingström, 1976). In addition, to increase pine growth and productivity, during the second half of the 20^th^ century, Contorta pine (*Pinus contorta*) was widely introduced in northern Sweden from Canada, where it is host to the local rust *M. medusae* (Elfving et al., 2001).

*Melampsora* pathogens have five sporulation stages in their life cycle tied to two alternate hosts (Newcombe and Chastagner, 1993; Mattila et al., 2001; Pei et al., 2005; Feau et al., 2009; Hacquard et al., 2013; Li et al., 2016). In temperate and boreal forests, a salicaceous host (poplar or willow depending on the rust species) becomes infested with dikaryotic (n+n) aeciospores. Throughout the growth season, the aeciospores develop and vigorously multiply on the leaves, initially as orange urediniospores (n+n) that subsequently become melanized “early (n+n)” and then (on senesced leaves) “late (2n)” teliospores. The teliospores are rich in lipids and glycogen, which enables the pathogen to tolerate winter frost (Feau et al., 2009). After undergoing karyogamy and meiosis, haploid basidiospores (n) are formed, which can then infect the alternate host (which may be a coniferous tree). Spermatogonia containing spermatia spores are then formed on the alternate host. The spores finally cross-fertilize to form dikaryotic (n+n) aeciospores that can infest another Salicaceae host (Hacquard et al., 2013).

Biomonitoring is essential for identifying and implementing appropriate management measures to control the build-up and spread of native and foreign pathogens (Choi and Park, 2019). Landscape-level information on abiotic and biotic damage to forests is therefore collected across Sweden by monitoring randomly selected individual trees and is made accessible through the Swedish National Forest Damage Inventory (SNFDI) database (Wulff et al., 2006). The biomonitoring data are based on visual scores that can be calibrated against spectral areal images (Roberge et al., 2016). Visual scores are also frequently used to assess resistance properties in common garden experiments (Newcombe and Chastagner, 1993; Albrectsen et al., 2010a; Robinson et al., 2012). Additionally, molecular detection and quantification techniques are increasingly used to analyze individual trees or small groups of trees (Feau et al., 2009; Ioos et al., 2010; Tan et al., 2010). Fungi share a conserved ITS rDNA sequence that can be amplified using specific primers, enabling PCR-based molecular verification and quantification of fungal infestations (Bourassa et al., 2005; Husson et al., 2013; Boutigny et al., 2013a; Boutigny et al., 2013b; Bergeron et al., 2019; Siddique et al., 2022). The *Melampsora* genus includes several species that use *P. tremula* as summer host including *M. medusae, M. larici-populina*, which are exotic in Sweden, and *M. pinitorqua*, which is native to Sweden. *M. medusae* and *M. larici-populina* have not been reported from northern Sweden, but since both pathogens are compatible with *Populus tremula*, we hypothesized that they could occur in northern Sweden for different reasons: *M. medusae* introduced by forestry from Canada along with fast-growing *Pinus contorta* and *M. larici-populina* through spread from distant populations of larch, potentially due to climate change. Primer pairs to detect these species are available (e.g., Bergeron et al., 2019) or can be designed using sequence data from sources such as NCBI GenBank (EU808032.1).

The aim of this study was to establish the relationship between visual rust scores and the presence of rust in leaves of aspen trees. First, we asked if the mycobiome marker genes in aspen leaves agreed among specific DNA coding for the *Melampsora* genus and for those of three rust species: the native *M. pinitorqua* and two exotic rusts (*M. medusae*, and *M. larici-populina*). Second, we asked if the impact of host genotype affected the relationship between visual rust scores and fungal DNA in the leaves. Third, we asked if expressed or genotype specific condensed tannin representations could explain the relationship between visual rust scores and the *Melampsora* representations.

## 2 Materials and methods

### 2.1 Biological materials and visual rust scoring (VRS) in the field 

In 2021, leaves were sampled from eleven-year-old aspen trees (Populus tremula). The trees grew in the “TanAsp common garden”, which was established in Vindeln, VB, Sweden, with genotypes representing extremes of the range of CT accumulation (high-CT: genotypes 5, 65, 72 and low-CT: genotypes 50, 60, 115; Bandau et al., 2015; Decker et al., 2017), within the founder SwAsp collection of 116 genotypes (Luquez et al., 2008). The garden included a total of 300 trees of which 72 were sampled for this study with 12 individuals per genotype. In 2021, the trees were up to 550 cm tall and leaf samples were collected at breast height on August 26, 2021. Five leaves were harvested randomly from each canopy and included both sun exposed and shaded leaves.

**Figure 1:**
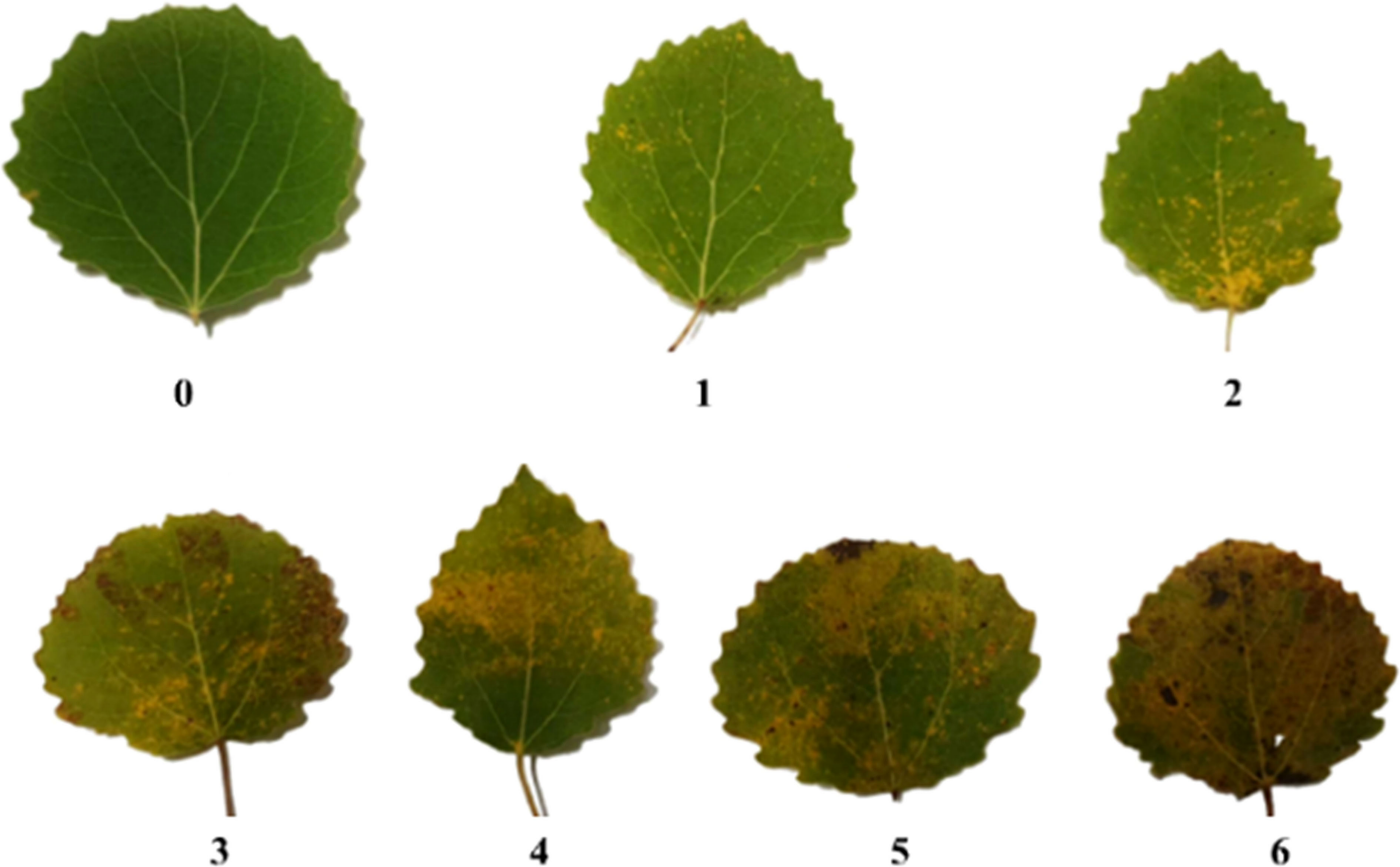
Visual scoring of infestation levels based on the density of rust pustules on a leaf. The visual rust score (VRS, **Table 1**) scale defines seven infestation levels (0 to 6) corresponding to 0, ~10, ~20, ~40, ~60, ~80, and 100% of the leaf area being covered with rust pustules, respectively.

As described by Siddique et al. (2022), leaves were placed in labeled and sealed plastic bags, flash-frozen in the field on dry ice, and transported to the lab in a cooler (Adiatic 24 L). The transportation process took around 1 h. The bags were then stored at -80°C in a freezer prior to lyophilization for 24 h in a freeze dryer (LABOGENE; 3450 Lillerød, Denmark) at a pressure of 0.02 mbar and a temperature of 108°C. The freeze-dried leaves were then ground individually to a fine powder using a mortar and pestle for downstream analysis. Leaves were always handled while wearing sterile gloves (Nitrile Ambidextrous Gloves, Thermo Fisher Scientific, Göteborg, Sweden) to prevent contamination with foreign DNA, and containers were sterilized between sample handling events. Before DNA extraction, each leaf was visually scored for rust using an index ranging from 0-6, indicating respectively that 0%, 15%, 30%, 45%, 60%, 75%, and 90% of the leaf surface exhibits symptoms of rust infestation (**Figure 1**).

### 2.2 DNA extraction and quality check

E.Z.N.A. Plant DNA kit (OMEGA BIO-TEK Inc., PW, Norcross, GA, USA) was used for fungal DNA extraction from single leaves, representing each one of the sampled trees, and as we failed to extract DNA from one of the leaves, the molecular work for this study was conducted on only 71 leaf samples. Following the provider’s protocol, 30 mg of leaf powder was used in each extraction. DNA was eluted from the mini columns with 50 µl elution buffer and then stored in a 1.5 ml Eppendorf tube at -20 °C. The concentration of each DNA extract was measured using a Nanodrop ND-1000 spectrophotometer (Thermo Fisher Scientific, Wilmington, DE 19810, USA) at wavelengths of 260/280 and 260/230 nm.

### 2.3 Primers and PCR

The fungal community load in each sample was determined by amplifying the conserved ITS2 region using a general primer pair (**Table 1**, Siddique et al., 2022). Four additional primer pairs were then used to amplify marker sequences specific to the *Melampsora* genus, the native pine twisting rust *M. pinitorqua*, and the two exotic rusts: *M. medusae* and *M. larici-populina* (Bergeron et al., 2019). The primers for *M. pinitorqua* amplification were designed using the primer3 software (Köressaar et al., 2018). In all cases, 25 µL PCR reactions were prepared using 2.5 µL Dream Taq DNA Buffer, 0.5 µL dNTPs (8 mM), 0.5 µL forward primers (10 mM), 0.5 µL reverse primers (10 mM), 0.16 µL Dream Taq DNA Polymerase, 1 µL template DNA, and 19.84 µL ultrapure deionized water. The PCR protocol was adjusted by changing the amount of template DNA, the annealing temperature and duration, and the number of PCR cycles. In all cases, PCR products were visualized under UV light after gel electrophoresis in a 1% agarose gel containing GelRed^®^ (Biotium, Inc., Fremont, CA, USA) (3 µL/100 mL agarose gel) at 140 volts for 30 min. The concentrations of PCR products were also measured using a Qubit Fluorometer (Thermo Fisher Scientific, Wilmington, DE 19810, USA), and the resulting values were used in subsequent analyses as indicators of fungal abundance.

**Table 1:** Primers used in this work to amplify the ITS2 region (representing the global fungal community), pathogenic rusts belonging to the genus Melampsora (*M.* spp.), and individual native (M. pinitorqua) *and non-native* (M. medusae and M. larici-populina) *rust species in* leaves of aspen (*Populus tremula*).

Primer name	Sequence (5´-3´)	Amplicon length (bp)	Specificity	References
ITS3F ITS4R	GCATCGATGAAGAACGCAGC TCCTCCGCTTATTGATATGC	250-400	Global fungal community	Siddique et al., 2022
MEL40F MEL40R	CCTGGTACTCCAACTATCATCTTA GAAWGTGCACGCGATTGACG	131	*Melampsora* spp.	Bergeron et al., 2019
MEL100F MEL100R	CACGAAAGTCBCAAGTGGC GTRCAGTCATGAGGTACGATA	148		
MEL176F MEL176R	GCCCTTGCCGTTGCTAT GRCTCGTGCTGATCAGTC	112		
MELpinF1 MELpinR1	GGTGCATTGTGGCCTTTCAC CAAACAGGCGTACCTTTCGG	282	*M. pinitorqua* *(GenBank: EU808032.1)*	This study.
MELpinF2 MELpinR2	GAGGTGCATTGTGGCCTTTC AAACAGGCGTACCTTTCGGA	283		
MM53F MM53R	ACAACCAGGTGACGGAAATC GAATCGTCCGAGGAGTCATT	129	*M. medusae*	Bergeron et al., 2019
MM74F MM74R	CACCATGCAAATCACCAATCAC TTTGGCTCAGCCTCAGTTTT	118		
MLP104F MLP104R	CGGCCAGAAATTGTGATGGAT TGCATAGCCTTTGTGGACAG	104	*M. larici-populina*	
MLP133F MLP133R	ATGGACCGGGAATATGAAC TCGTTGATCGTATCGTGGAA	126		

### 2.4 Condensed tannin assay

Soluble CTs were analyzed using the acid-butanol method according to Porter et al. (1986), and as described in Bandau et al. (2015). Briefly, 10 ± 2 mg leaf powder was dissolved in 1 mL of acetone solution (70% acetone with 0.01% ascorbic acid) and centrifuged at 15,000 rcf. A 50 µL aliquot of the supernatant was then transferred to 600 µl of butanol-HCl solution containing 20 µl of the iron reagent (2% ferric ammonium sulfate in 2N HCl). After incubation, the absorbance was measured in three technical replicates and compared to a standard curve of procyanidin B2 (C_30_H_26_0_12_, Sigma-Aldrichl, St. Louis, MO, USA) for quantification. Absorbance was measured at 550 nm on a Hitachi U-5100 UV/VIS spectrophotometer (Hitachi High-Technologies, Tokyo, Japan). Concentrations of soluble CTs are reported in units of mg/g DW.

### 2.5 Statistical analysis

The effects of genotype, nitrogen treatment history, and their interaction were evaluated using ANOVA, non-parametric tests, and the χ^2^ test depending on residuals, normality, and data type. R (version 4.2.1) was used to perform all statistical analyses and to generate all the associated graphs and figures.

## 3 Results

### 3.1 PCR-based detection of *Melampsora* spp.

**Figure 2:**
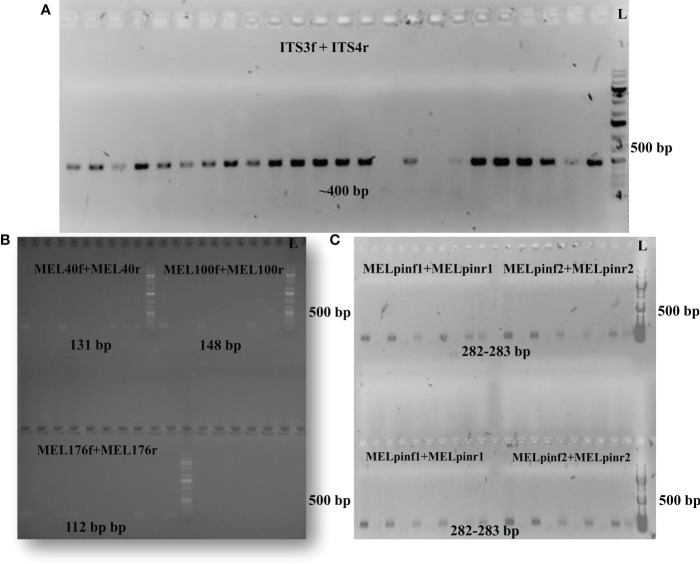
Gel images confirm the presence of DNA from targeted taxa. **(A)** Strong bands representing the conserved ITS2 region (using ITS3f+ITS4r primer pairs) confirmed the presence fungal rDNA. **(B)** Amplifications using the MEL40, MEL100 and MEL176 primer pairs detected DNA of the *Melampsora* genus. **(C)** Amplifications using the MELpin1 primer pairs with annealing temperatures of 58°C (upper panel) and 60°C (lower panel) confirmed the presence of *M. pinitorqua.* DNA ladder (L) indicates the size of the amplified PCR products.

Protocol optimization (**Table 2**) resulted in the development of a reliable method for quantifying the overall fungal load (i.e., the total mycobiome) in aspen leaves by PCR targeting the conserved ITS2 region of fungal rDNA. The abundance of the genus *Melampsora* spp. could also be determined using three previously described primer pairs (Bergeron et al., 2019). The native *M. pinitorqua* was amplified using primer pairs developed for this study (**Tables 1**, **2**). Bergeron et al. (2019) also developed primers targeting the exotic rust species *M. medusae* and *M. larici-populina*, but experiments using these primers yielded no evidence that either species was present in the samples (**Table 2**). The observation of gel bands at ~400 bp confirmed the presence of fungal species and *Melampsora* taxa in TanAsp genotypes with no evidence of non-autochthonous rust interactions in the samples (**Table 2**; **Figures 2A–C**).

**Table 2:** Results of PCR amplifications of DNA originating from microbial fungi in leaves from six aspen genotypes grown in the TanAsp common garden in Vindeln, Sweden.

Targeted taxa	Primer pairs	PCR cycle number	Annealing temperature (°C)	Template DNA (µl)	Result
*Mycobiome (ITS2)*	ITS3F + ITS4R	35	57	1	Strong band (**Figure 2A**)
*Melampsora spp*	MEL40F + MEL40R	35	58	1 2	No band
		35	60	1 2	Medium- strong band (**Figure 2B**)
	MEL100F+MEL100R	35	58	1 2	No band
		35	60	1 2	Medium- strong band (**Figure 2B**)
	MEL176F+MEL176R	35	58	1 2	No band
		35	60	1 2	Medium band (**Figure 2B**)
*Melampsora medusae*	MM53F + MM53R	35	58	1 2	No band
			60	1 2	No band
		37	58	1 2	No band
			60	1 2	No band
		38	58	1 2	No band
			60	1 2	No band
	MM74F + MM74R	35	58	1 2	No band
			60	1 2	No band
		37	58	1 2	No band
			60	1 2	No band
		38	58	1 2	No band
			60	1 2	No band
*Melampsora larici-populiina*	MLP104F + MLP104R	35	58	1 2	No band
		37	58	1 2	No band
		38	58	1 2	No band
	MLP133F + MLP133R	35	58	1 2	No band
		37	58	1 2	No band
		38	58	1 2	No band
*Melampsora pinitorqua*	MELpinF1 + MELPinR1	35	58	1	Strong band (**Figure 2C**)
		35	60	1	
	MELpinF2 + MELPinR2	35	58	1	
		35	60	1	

Primer pairs targeting the conserved ITS2 region were used to examine the entire mycobiome, while genus-specific primers targeting Melampsora spp and species-specific primers targeting markers of non-native (M. larici-populina, M. medusae) and native (M. pinitorqua) were used to characterize rust infestations.

### 3.2 Effects of aspen host genotype

The TanAsp common garden was established in 2010 and contains aspen genotypes representing extremes of the species’ range of CT accumulation behavior. Trees in the garden were initially subjected to fertilization with nitrogen (Bandau et al., 2021). These historical nitrogen treatments had no detectable effect on the burden of fungal DNA in the leaves or any other rust-related responses examined in this work, either as single effects (**Supplementary Material S1**) or as genotype interactions (**Supplementary Materials S2, S3** **)**. The effect of historic nitrogen addition was therefore disregarded in all subsequent analyses.

Genotype-specific responses were observed for visual rust scores (VRS) and the abundance of marker sequences from the *Melampsora* genus. However, host genotype had no detectable effect on the abundance of genetic markers of the general mycobiome or *M. pinitorqua*, or on the CT concentration in the leaves (**Table 3**).

**Table 3:** Test values measuring the effects of aspen genotype on foliar rust related responses including visual rust scores determined in the field (VRS, **Figure 1**), foliar concentrations of Condensed Tannins (CTs, mg/g DW), and molecular markers representing the overall fungal load (ITS2) as well as the abundance of fungi belonging to the *Melampsora* genus (using the MEL40 and 100 primers) and the native rust *M. pinitorqua* (using the MELpin1 primers).

Responses	Test-value	P - Values
VRS	χ2 = 78.40	1.9 e-07 ***
CTs	F* =1.51	0.19
ITS2	H = 8.41	0.14
MEL40	F = 2.90	0.02*
MEL100	F = 3.11	0.014*
MELpin1	H = 5.24	0.39

Tests were chosen based on data type and test assumptions (**Figure 2**). The chi-squared (χ2) test was used for count data, parametric ANOVA was used for continuous data (F) with normally distributed residuals (with log transformation applied in one case, indicated by the symbol F*), and non-parametric Kruskal-Wallis tests (H) in cases where the normality criterion was not satisfied. *p < 0.05, ***p < 0.001.

**Figure 3:**
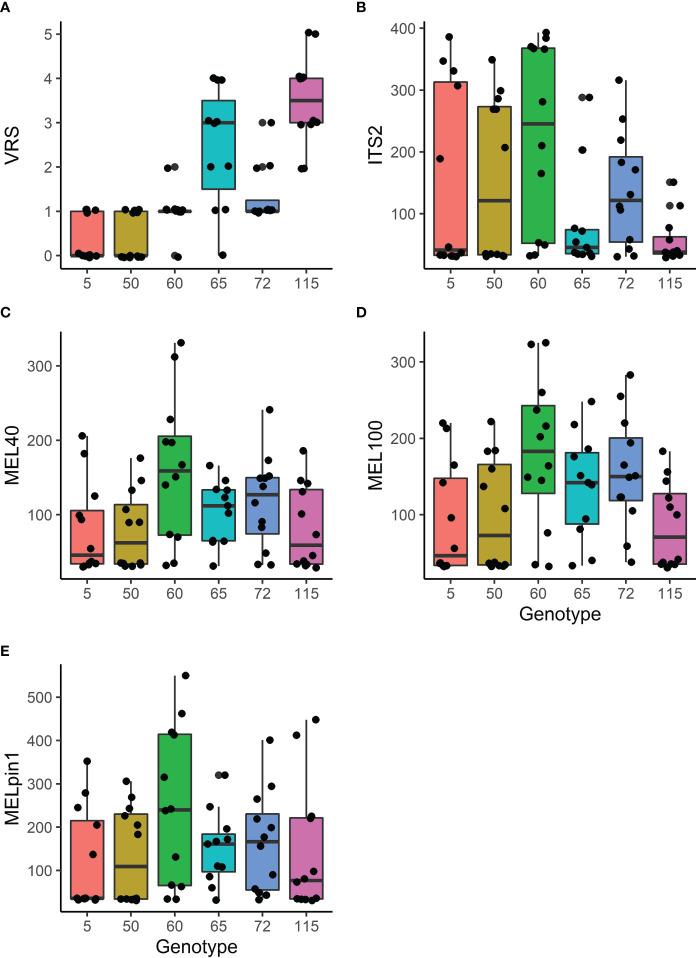
The effect of genotype on foliar rust related responses: **(A)** Visual rust score (VRS; for details, see **Figure 1**). **(B)** PCR concentration (ng/ml) of molecular markers of general fungal presence (ITS2 region of fungal rDNA amplified using ITS3f+ITS4r); **(C, D)** Fungal DNA originating from the *Melampsora* genus (MEL40 and MEL100); and **(E)** Fungal DNA from the native rust *M. pinitorqua* (MELpin1) (see also **Table 1**). Test summaries are given in **Table 3**. The upper and lower boundaries of the boxes correspond to the 25^th^ and 75^th^ percentile values, respectively, and the horizontal lines inside the boxes indicate the median value for the relevant genotype. Vertical lines represent the range of values observed per tested unit based on 11 or 12 replicates per genotype and the black dots are the representation of individual observations.

The VRS scores of the genotypes differed significantly; genotypes 5 and 50 had very low VRS scores (indicating minimal evidence of infestation) whereas genotypes 65 and 115 had clearly visible severe infestations (**Figure 3A**). Interestingly, the ranking of the genotypes with respect to ITS2 sequence abundance appeared to be the inverse of that for VRS scores (**Figure 3B**), although the differences in ITS2 abundance were non-significant (**Table 3**). Two primer pairs (MEL40 and MEL100) were used to amplify a genetic marker of the genus *Melampsora*, and both achieved similar levels of amplification (**Figures 3C, D**).

Amplifications using the primer pair MELpin1, which is specific to the species *M. pinitorqua*, revealed no strong effect of aspen genotype on the abundance of this species, although its abundance was relatively high in aspen genotype 65. The ranking of the aspen genotypes based on *M. pinitorqua* abundance was intermediate between the VRS rankings and the ITS2 rankings (**Figure 3E**; **Table 3**).

### 3.3 Relationship between VRS and molecular rust indications

**Figure 4:**
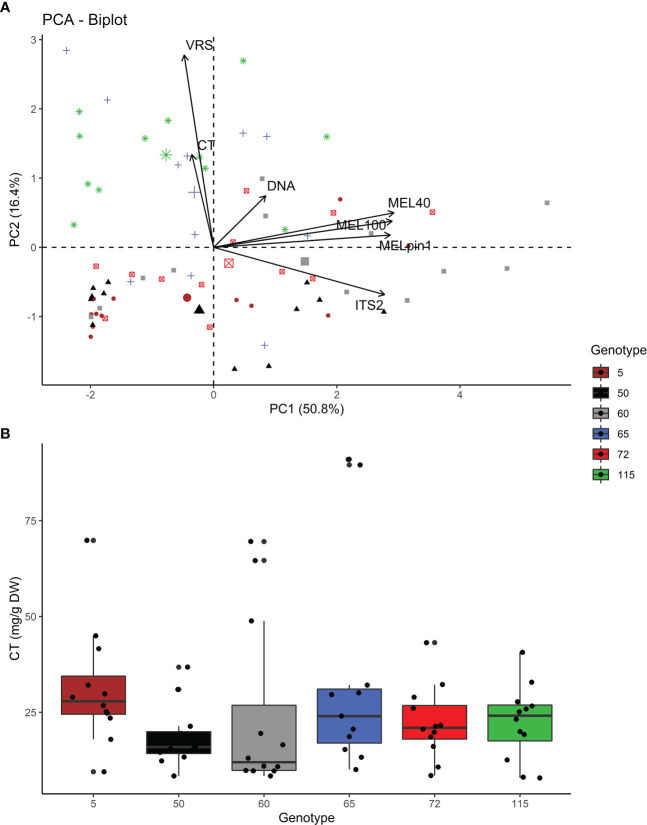
**(A)** PCA biplot showing the relationships between the studied indicators of fungal infestation at genotypic level: VRS (visual rust score), foliar condensed tannin content (CT), DNA concentration, and the PCR concentration (ng/ml) for the marker genes of general fungal colonization (the ITS2 fungal rDNA sequence amplified using ITS3+ITS4 primers), the *Melampsora* genus (amplified using the MEL40 and MEL100 primers), and the native rust *M. pinitorqua* (amplified using the MELpin1 primers). Colors and shapes represent the observation for individual genotypes. Correlations between the responses are shown in **Figure 5**. **(B)** Foliar concentrations of condensed tannins (CT) in the six studied aspen genotypes and expressed as mg/g dry weight (mg/g DW).

**Figure 5:**
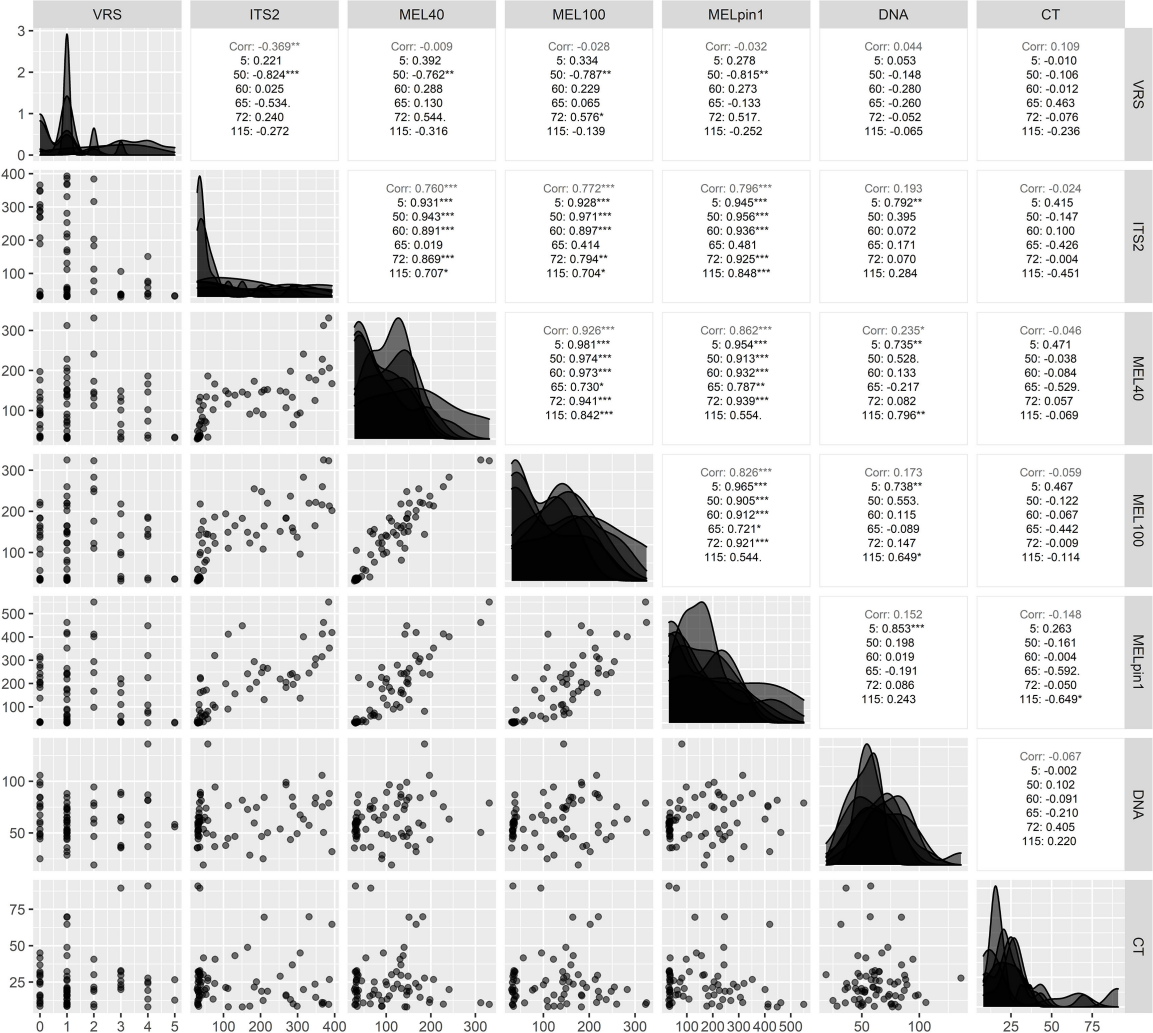
Correlations between the VRS scores of leaves from six aspen genotypes and their contents of four molecular markers of fungal abundance: ITS2 (representing the total mycobiome), MEL40 and MEL100 (representing PCR concentration of marker genes for the genus *Melampsora*), and MELpin1 (representing PCR concentration of marker gene for the native rust *M. pinitorqua*). Data are presented in a diagonalized manner: the left-hand triangle show scatterplots visualizing the pairwise relationships between the responses, while the right-hand triangle show the Pearson correlation coefficients between the responses for both the full set of samples and for each genotype individually. The graphs on the diagonal show the distribution of the indicated response within each genotype. *p < 0.05, **p < 0.01, ***p < 0.001.

As expected, correlational differences were detected among rust related responses. A PCA biplot (**Figure 4A**) revealed a positive relationship between the presence of *Melampsora* at the genus level (with MEL40 and MEL100 primer pairs) and the species-specific presence of *M. pinitorqua* (MELpin1), which was supported by high positive Pearson correlation coefficients ranging from ρ=0.826*** to ρ=0.926*** (**Figure 5**). The abundance of *Melampsora* DNA also correlated strongly with that of the overall mycobiome (PCR concentration of ITS2 region amplified using the ITS3f+ITS4r primer pairs), giving correlation coefficients of ρ = 0.76*** to ρ=0.796*** (**Figure 5**). Conversely, the amplicon abundance obtained using the MEL-specific primers did not correlate strongly with the samples’ total fungal DNA content (ρ = 0.152^n.s^ to ρ=0.235*, **Figure 5**). All the pairwise correlations exhibited pronounced genotype dependence (**Figure 5**), replicating the pattern seen in the univariate genotype rankings (**Figure 3**). Interestingly, the visual rust score (VRS) did not correlate well with any molecular *Melampsora* indicators (ρ = -0.009, -0.028, and -0.032 for MEL40, MEL100, and MELpin1 respectively). The VRS also correlated negatively with the ITS2 sequence abundance (-0.369**), indicating a negative association with overall fungal abundance. All these correlations were strongly genotype-dependent, as demonstrated by the genotype-specific correlations shown in **Figure 5**.

### 3.4 Effect of leaf CTs on molecular representation of *Melampsora* and fungi in general

Unexpectedly, the data obtained revealed no strong effect of CT content on fungal abundance. Even though the genotypes in the common garden were chosen to represent extremes within the range of CT accumulation for aspen, they could not be separated based on the measured tannin contents of their leaves (**Table 3**). In addition, the associations between the samples’ CT concentrations and the rust molecular responses were uniformly negative and non-significant: the ρ values for the correlation with CT were -0.024 for ITS2, -0.046 for MEL40, -0.059 for MEL100, -0.067 for DNA, and -0.148 for MELpin1 (**Figures 4**, **5**). Similarly, the CT-groupings of the aspen genotypes were either not correlated with the rust molecular responses or exhibited weak negative relationships (**Supplementary Material S4**). This may be because CT accumulation is highly variable; the CT group membership (high or low) assigned to each genotype in the common garden was only related to the measured CT concentrations in the leaf samples by a trend (**Supplementary Material S5**, two-sided test F=3.19; p =0.08). However, a statistically significant relationship between CT group membership and the measured CT concentration was observed using a one-sided test (p=0.04).

## 4 Discussion

PCR-based identification of *Melampsora* taxa confirmed the presence of the native pine twisting rust (*M. pinitorqua*) in leaves of *Populus tremula* but not that of the exotic rusts *M. medusae* and *M. larici-populina*. Moreover, the abundance of *M. pinitorqua* (quantified using a Qubit fluorometer) correlated strongly with that of the *Melampsora* genus in the samples. A strong positive relationship was also suggested between *Melampsora*-specific primer products and the products of ITS2 region of fungal rDNA amplified with ITS3f+ITS4r primers, indicating that *Melampsora* rust may have dominated the fungal community during the sampling event. However, there was also strong evidence that the conditions for rust establishment were highly dependent on the aspen genotype.

Fungal communities in tree foliage are not uniformly distributed or stationary (Toju et al., 2019); their composition is influenced by several factors including host genotype (Albrectsen et al., 2010b), growth site (Siddique et al., 2021), seasonal factors (Albrectsen et al., 2010b), ontogeny (Bourassa et al., 2005; Boutigny et al., 2013a), tissue type (Siddique et al., 2021), and host-associated consumers (Arnold et al., 2003; Albrectsen et al., 2010b; Siddique et al., 2021). The mechanisms underpinning these relationships are not fully understood but it has been suggested that both the host’s chemical defense responses (Ullah et al., 2017; Witzell et al., 2022) and competition between members of the mycobiome may help shape the microbial community (Wemheuer et al., 2019; Siddique et al., 2021). In the present study, we also obtained results suggesting the occurrence of a transition in the composition of the fungal community. Interestingly the genotype with the highest load of Melampsora DNA had both intermediate visual rust scores (VRS) and an intermediate level of metagenomic fungal representation based on ITS2 amplification. However, these two traits also had opposing relationships with genotype. It may be that the VRS were influenced by ontogenetic factors because low scores (VRS level 1-3, **Figure 1**) mainly reflect the presence of orange urediniospores. As the infestation progresses, teliospores start developing. In addition, the uredinosporangia generate an uneven leaf surface with disrupted underlying leaf cells, which could facilitate colonization by decomposing epiphytes. This may be why the observed relationship between genotype and ITS2 DNA abundance was opposed to that for genotype and VRS. The high amplification of *Melampsora*-related DNA in certain genotypes (particularly genotype 60) could thus be due to a combination of these ontogenetic processes. Overall, our results suggest that while VRS may be useful indicators of rust incidence in a stand, it does not appear to accurately predict *Melampsora* DNA loads. Pathogen virulence depends on the three factors comprising the disease triangle, namely the pathogen, host, and environment. However, in the case of rust, the existence of an alternate host creates complex relationships among these factors, and visible rust symptoms on aspen trees do not seem to be reliable indicators of twisting rust abundance (and thus of risks to adjacent pine trees).

Identification, detection, and quantification of disease-causing organisms are all important for biomonitoring of native and forest pathogens. Approaches used to detect and monitor pathogens include scoring of morphological symptoms, culture-based studies, and microscopic identification, all of which have their limitations (Husson et al., 2013; Boutigny et al., 2013b). PCR-based molecular techniques can detect and quantify disease-causing agents directly by amplifying their DNA (Boonham et al., 2008; Sundelin et al., 2009; Pothier et al., 2011; Husson et al., 2013; Boutigny et al., 2013a; Boutigny et al., 2013b; Yu et al., 2019; Avenot et al., 2022), and have been particularly successful in targeting and detecting known pathogens (Bourassa et al., 2005; Husson et al., 2013; Boutigny et al., 2013b; Guinet et al., 2016; Bergeron et al., 2019). Accordingly, we successfully used PCR to confirm the presence of a mycobiome and *Melampsora* taxa in the studied leaf samples. However, the exotic rusts *M. medusae* and *M. larici-populina* were not detected in our PCR experiments, although it should be noted that such negative results cannot be considered confirmatory (Sober, 2009). PCR-based approaches also have their downsides; notably, they only amplify a small region of a marker gene. This may limit their resolution because the targeted sequence may be common to multiple taxa, potentially leading to false identifications resulting from mismatches between the chosen primer pairs and the organism being targeted. Such problems can be overcome in part by using high throughput sequencing methods (Lorrain et al., 2018) and the risk of detection errors can be further reduced by RNA sequencing (Yu et al., 2019; Avenot et al., 2022).

Aspen is rich in phenolic defense compounds including condensed tannins (CTs), which have been negatively associated with endophytic communities (Bailey et al., 2005), the aspen pathogen Venturia (Bandau et al., 2021), and rusts (Walkinshaw, 1989). We therefore expected negative associations between foliar CT levels and fungal markers. Unexpectedly, we instead observed a positive relationship between VRS and CTs as well as weak negative correlations between CTs and the studied molecular fungal markers. While these patterns might initially appear to be inconsistent, they might be partly explained by the dynamic nature of CTs and the responses of rust to an impermeable CT barrier. CTs are concentrated in the epidermal layer of leaves (Rodríguez et al., 2022, supporting information S3), where they provide a barrier to pathogen entry. However, they do not restrict the spread of spores on the leaf surface, where multiple infestation attempts would trigger hypersensitivity responses (HR) resembling the later teliospore stages of rust infestation. CT-rich leaves could thus exhibit apparent visual symptoms of severe infestation on the surface even in the absence of such infestation. Consequently, plants exhibiting these symptoms would not have a high abundance of *Melampsora* DNA and would present no great risk of infestation to adjacent pine trees. Moreover, in late summer (when sampling was performed in this study), aspen leaves will have been exposed to both abiotic and biotic stress factors. Several biotic factors cause the degradation of CTs into catechin and reactive oxygen species (Ullah et al., 2017; Gourlay and Constabel, 2019), which may explain why the sampled trees did not have unusually high or low CT contents even though the genotypes in the common garden were selected to represent extremes in the natural range of CT production within aspen (Bandau et al., 2015; Bandau et al., 2021). The dynamic behavior of CTs has made it difficult to understand their function. While early studies assumed that CTs play a general protective role (Zhang et al., 2009; Yang et al., 2018; Tong et al., 2021), a growing body of evidence suggests that it is actually this dynamic behavior that is responsible for their defensive effects, and that a tree’s dynamic range or amplitude of CT-reactivity may be the real driver of its tannin-based tolerance of and resistance to pathogens.

Large-scale herbicide treatment is no longer practiced in modern Swedish forestry; instead, current efforts to avoid pine twisting damage focus on management interventions to support healthy pine trees. *Pinus contorta* is highly resistant to *M. pinitorqua* and we previously found no evidence that the *M. medusae* complex had accompanied lodgepole pine from Canada when it was introduced to Sweden around 60-70 years ago (Witzell, 2017). This finding is tentatively supported by the study reported herein, in which only autochthonous rust relationships were identified.

## 5 Conclusion

Overall, our results support the rust control strategy adopted by the Swedish Forest Agency, which focuses on resistance in pine regeneration rather than attempting to act on rust symptoms in aspen trees. While rust scoring may provide information on the incidence of rust within a forest stand, our results do not support the use of visual rust scores to assess epidemiological parameters such as infestation risk. Our findings also suggest that *Melampsora* rust on aspen in Sweden is dominated by the native and autochthonous pine twisting rust, and that exotic rust species associated with Lodgepole pine and larch are highly unlikely to have invaded northern Sweden.

## Data availability statement

The original contributions presented in the study are included in the article/**Supplementary Material**. Further inquiries can be directed to the corresponding author.

## Author contributions

AS: Formal analysis (lead); project administration (lead); validation (lead); visualization (lead); writing—original draft (supporting); writing—review and editing (supporting). LM, MD: Laboratory work (supporting); writing—original draft (supporting); writing—review and editing (supporting). BA: Conceptualization (lead); data curation (supporting); formal analysis (supporting); funding acquisition (lead); investigation (lead); methodology (lead); project administration (lead); resources (lead); software (supporting); supervision (lead); validation (supporting); visualization (supporting); writing—original draft (lead); writing—review and editing (lead). All authors contributed to the article and approved the submitted version.
