# Supplementary material for: Molecular studies of rust on European aspen suggest an autochthonous relationship shaped by genotype
Source: Front Plant Sci. 2023 Feb 20;14:1111001. doi: 10.3389/fpls.2023.1111001 (PMC9986475; doi:10.3389/fpls.2023.1111001)
Supplement: Supplementary file 2 [file DataSheet_2.docx]

**Supplementary Materials**

**
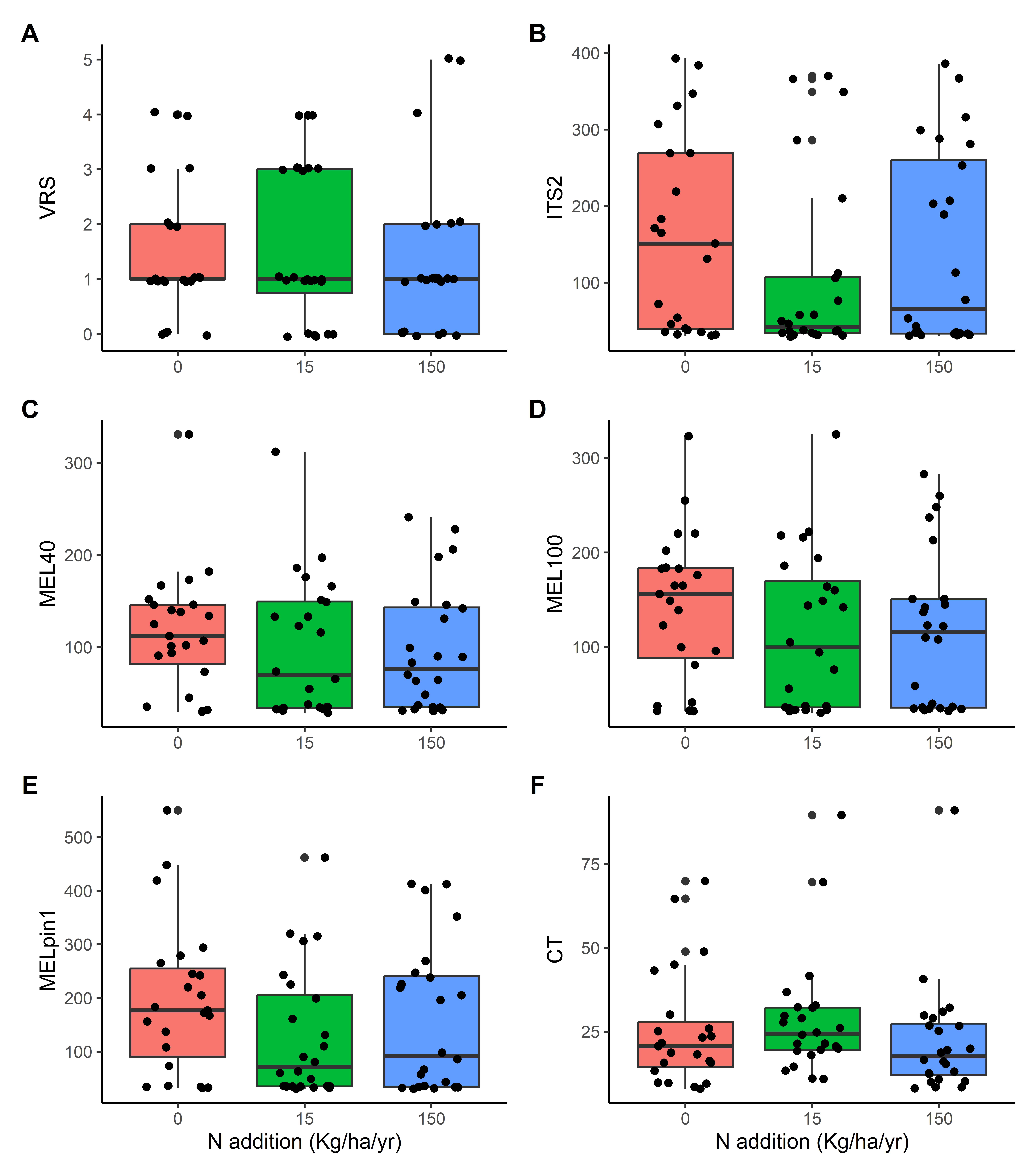
**

**Figure S1**. Boxplots showing the effects of historical nitrogen addition (Bandau et al 2021) on indicators of rust infection including **A** A visible rust scores (VRS) scores based on field observations, **B** PCR amplification of the ITS2 marker of the global fungal community, **C and D** PCR amplification using primers specific for the *Melampsora* genus, **E** PCR amplification using primers specific for *M. pinitorqua*, and **F** the measured concentrations of condensed tannins in the sampled leaves. The upper and lower boundaries of the boxes correspond to the 25^th^ and 75^th^ percentile values, respectively, and the horizontal lines inside the boxes indicate the median value for the relevant genotype. Vertical lines represent the range of values observed per tested unit (n=24). Each observation is represented by a dot.


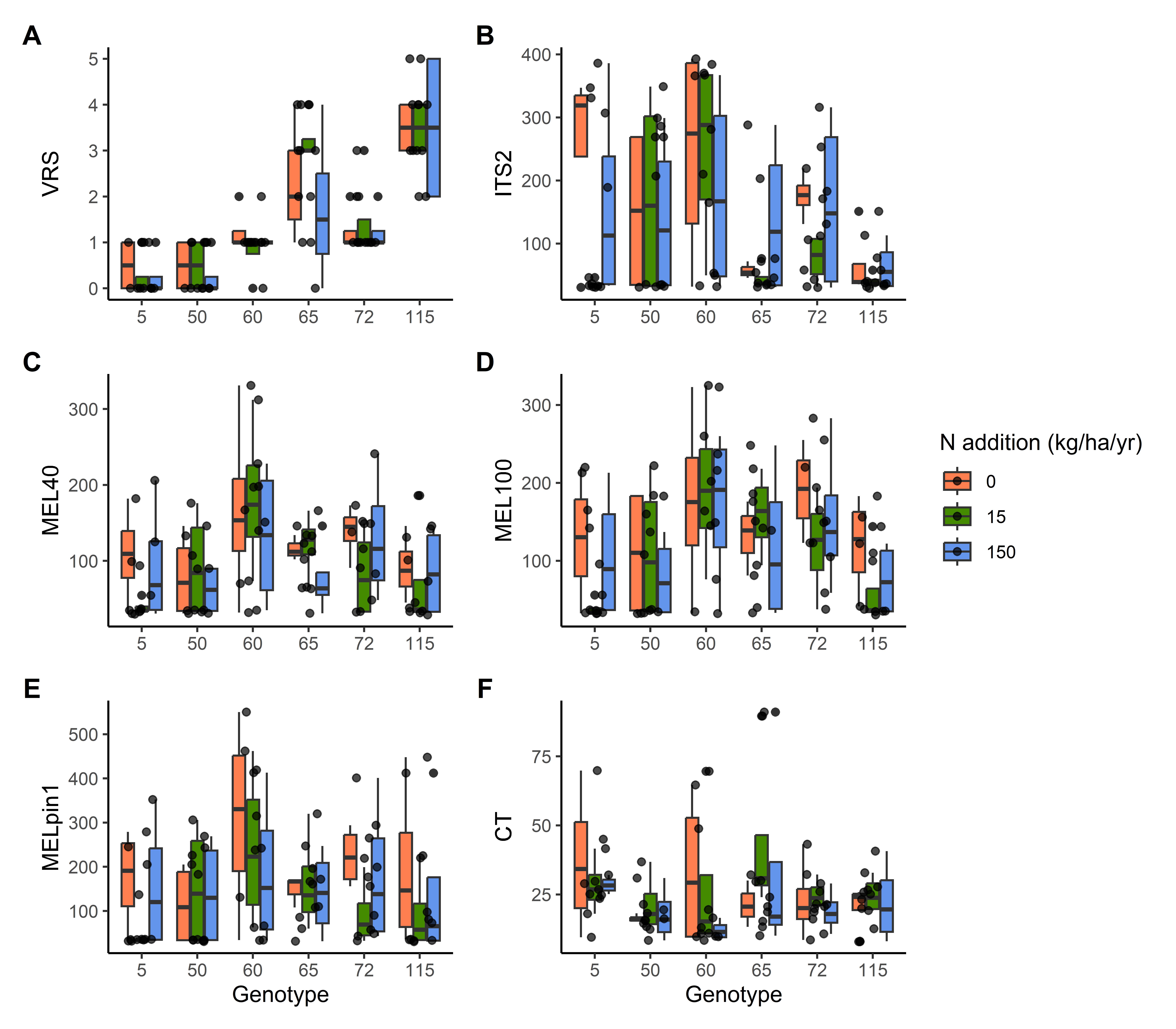


**Figure S2.** Boxplot showing the interactive effect of TanAsp genotype and nitrogen amendment on rust infection measured both phenotypically and by PCR targeting genetic markers of the global fungal community, *Melampsora spp*, and *M. pinitorqua*. The upper and lower boundaries of the boxes correspond to the 25^th^ and 75^th^ percentile values, respectively, and the horizontal lines inside the boxes indicate the median value for the relevant genotype. Vertical lines represent the range of values observed per tested unit (n=4).

**Table S3**. ANOVA results for analyses of the effect of genotype, nitrogen amendment history, and their interaction on rust related responses in aspen leaves. The responses include the visual rust score based on field observations (VRS, Figure 1), leaf tannin (CT) status at harvest (as specified in Table 1), and DNA concentrations obtained from PCR amplifications targeting general fungal ITS markers, genus-specific markers (*Melampsora spp*., MEL40 and MEL100), and species-specific markers (*Melampsora pinitorqua,* MELPin1 and MELPin2).

| Responses | Genotypes | | Treatment | | Genotype*Treatment | |
| --- | --- | --- | --- | --- | --- | --- |
|  | F | P | F | P | F | P |
| VRS | 26.71 | 2.24e-13 | 0.82 | 0.44 | 0.64 | 0.77 |
| ITS | 3.07 | 0.02 | 1.43 | 0.25 | 0.84 | 0.60 |
| MEL40 | 2.66 | 0.03 | 0.63 | 0.54 | 0.54 | 0.85 |
| MEL100 | 2.86 | 0.02 | 0.97 | 0.38 | 0.49 | 0.89 |
| MELpin1 | 1.28 | 0.29 | 1.36 | 0.27 | 0.45 | 0.91 |
| CTs | 1.26 | 0.30 | 0.77 | 0.47 | 0.53 | 0.86 |

**
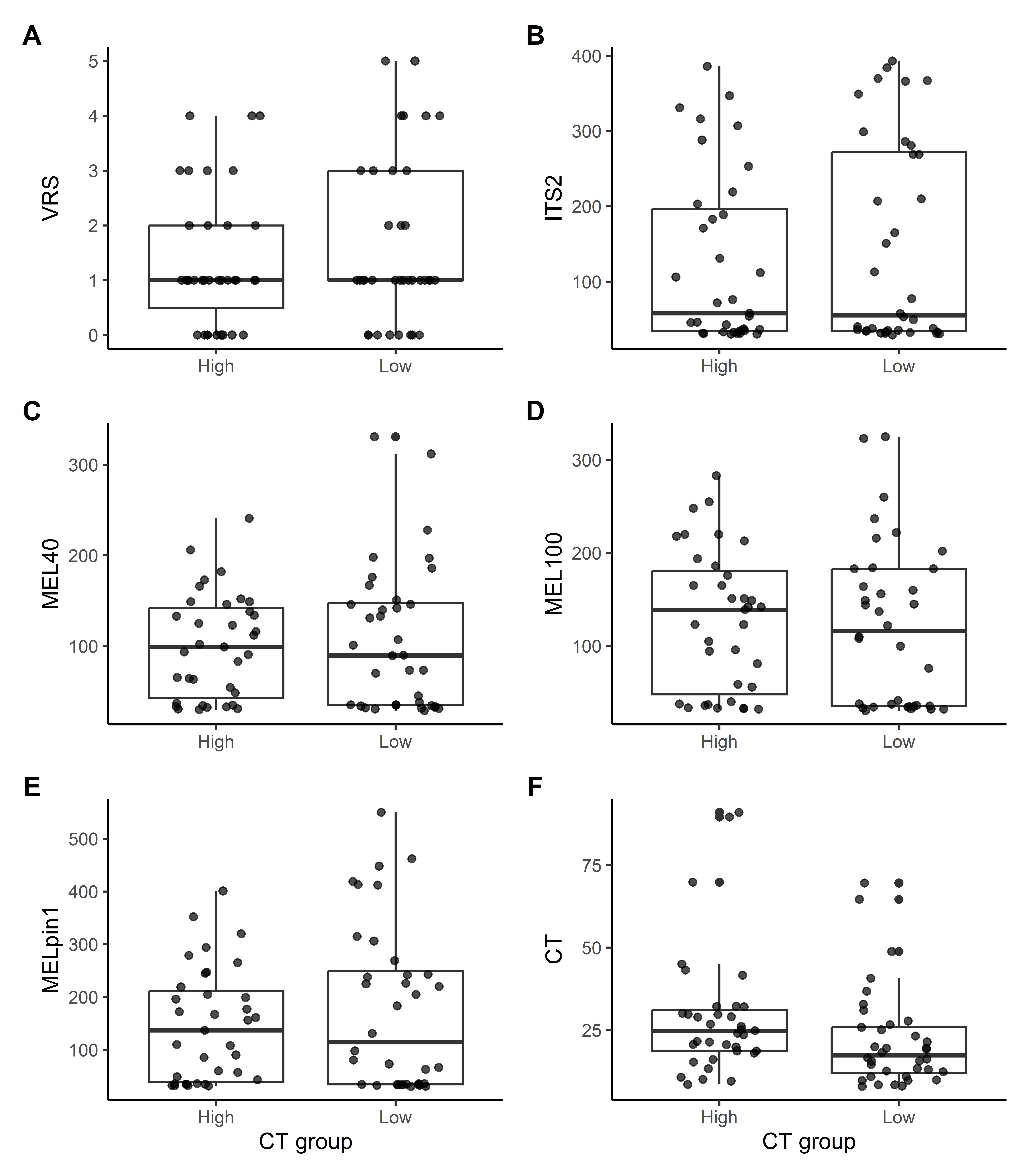
**

**Figure S4.** Boxplots showing the effects of condensed tannins group membership (Bandau et al., 2021) on the following foliar rust related responses: **A** visual rust score (VRS, scores range from 0 to 6 as shown in Figure 1). **B** PCR concentration (ng/ml) of molecular markers of general fungal abundance (ITS2), **C** and **D** markers of fungal DNA from the genus *Melampsora* (MEL40 and MEL100), **E** markers of fungal DNA from the native rust *M. pinitorqua* (MELpin1), and **F** measured foliar Condensed Tannins concentrations (CT, mg/gDW) (see also Table 1). Details of test statistics are provided in Table 3. The upper and lower boundaries of the boxes correspond to the 25^th^ and 75^th^ percentile values, respectively, the horizontal lines inside the boxes indicate the median value for the relevant genotype. The dots represent individual observations. Vertical lines represent the range of values observed per tested unit (35 or 36 replicates per genotype).

**Table S5**. ANOVA results for an analysis of the effect of Condensed Tannins group membership (Bandau et al., 2021) on rust-related responses in aspen leaves. The responses include the visual rust score based on field observations (VRS, Figure 1), leaf tannin (CT) status at harvest (as specified in Table 1), and DNA concentrations obtained from PCR amplifications targeting general fungal ITS markers, genus-specific markers (*Melampsora spp*., MEL40 and MEL100), and species-specific markers (*Melampsora pinitorqua,* MELPin1 and MELPin2).

| Responses | Test-value | P - Values |
| --- | --- | --- |
| VRS | χ2 = 2.33 | 0.80 |
| ITS2 | H = 0.16 | 0.68 |
| MEL40 | F = 0.19 | 0.67 |
| EL100 | F = 0.08 | 0.77 |
| MELpin1 | H = 0.06 | 0.80 |
| CTs | F* = 3.19 | 0.08 |
